# Transcriptome profiling revealed early vascular smooth muscle cell gene activation following focal ischemic stroke in female rats – comparisons with males

**DOI:** 10.1186/s12864-020-07295-2

**Published:** 2020-12-09

**Authors:** Mimmi Rehnström, Simona Denise Frederiksen, Saema Ansar, Lars Edvinsson

**Affiliations:** 1 Department of Internal Medicine, Lund University Hospital, S22185 Lund, Sweden; 2 Independent researcher, Calgary, Alberta Canada; 3 Department of Clinical Sciences, Neurosurgery, Lund University, Lund, Sweden

**Keywords:** Focal cerebral ischemia, Transcriptomics, mRNA, Gene regulation, Female rats, Sex differences, Transcription factors, Inflammation, Endothelial function, Pathway analysis

## Abstract

**Background:**

Women account for 60% of all stroke deaths and are more often permanently disabled than men, despite their higher observed stroke incidence. Considering the clinical population affected by stroke, an obvious drawback is that many pre-clinical and clinical studies only investigate young males. To improve therapeutic translation from bench to bedside, we believe that it is advantageous to include both sexes in experimental models of stroke. The aims of this study were to identify early cerebral vascular responses to ischemic stroke in females, compare the differential gene expression patterns with those seen in males, and identify potential new therapeutic targets.

**Results:**

Transient middle cerebral artery occlusion (tMCAO) was used to induce stroke in both female and male rats, the middle cerebral arteries (MCAs) were isolated 3 h post reperfusion and RNA was extracted. Affymetrix whole transcriptome expression profiling was performed on female (*n* = 12) MCAs to reveal differentially expressed genes. In total, 1076 genes had an increased expression and 879 genes a decreased expression in the occluded MCAs as compared with the control MCAs from female rats. An enrichment of genes related to apoptosis, regulation of transcription, protein autophosphorylation, inflammation, oxidative stress, and tissue repair and recovery were seen in the occluded MCA**.** The high expression genes chosen for qPCR verification (*Adamts4*, *Olr1*, *JunB*, *Fosl1*, *Serpine1*, *S1pr3*, *Ccl2* and *Socs3*) were all shown to be upregulated in the same manner in both females and males after tMCAO (*p* < 0.05; *n* = 23). When comparing the differentially expressed genes in female MCAs (occluded and non-occluded) with our previous findings in males after tMCAO, a total of 297 genes overlapped (all groups had 32 genes in common).

**Conclusions:**

The cascades of processes initiated in the vasculature following reperfusion are complex. Dynamic gene expression alterations were observed in the occluded MCAs, and to a less pronounced degree in the non-occluded MCAs. Dysregulation of inflammation and blood-brain barrier breakdown are possible pharmacological targets. The sample of genes (< 1% of the differentially expressed genes) validated for this microarray did not reveal any sex differences. However, sex differences might be observed for other gene targets.

**Supplementary Information:**

The online version contains supplementary material available at 10.1186/s12864-020-07295-2.

## Background

Ischemic stroke is one of the leading causes of death and disability in the world [1]. Although the incidence is higher in men, women account for 60% of all stroke deaths and are more often permanently disabled than men [2]. Thrombolysis, the only available non-invasive treatment for stroke, has in some studies been shown to have a better effect in women than in men [3, 4]. Although reperfusion by thrombolysis or thrombectomy has been shown effective in salvaging neurological function, restoration of blood flow and reduction of damages to the blood-brain barrier (BBB) increases the risk of hemorrhagic transformation and edema, which may be potentially fatal complications [5]. In the case of thrombolysis, the risk of these adverse effects does not outweigh the benefits past 4.5 h post stroke, which limits the use in clinical practice [6].

Despite intense research efforts during several decades with more than 1000 compounds tested and numerous interventions that have shown promise in pre-clinical studies, all failed in the clinical studies [7]. Some of the main reasons proposed for this “translational roadblock” in stroke treatment are related to the fact that the majority of pre-clinical studies have been performed in young healthy male rodents, a clear drawback when considering the clinical population affected by stroke. By including both sexes in experimental models of stroke, it may be possible to more accurately represent the clinical scenario and thus improve therapeutic translation from bench to bedside. Despite increasing awareness of the importance of sex differences, the majority of pre-clinical and clinical studies are still performed on males [8].

Developing effective treatment strategies for both men and women requires a deeper understanding of sex differences in the underlying mechanisms of ischemic injury. In experimental stroke models, female animals have smaller ischemic areas and better functional outcomes, and this difference is nullified by ovariectomy, suggesting that female sex hormones (estrogen and progesterone) are responsible [9]. The protective effect of estrogen has been shown to be multifactorial, acting on both the vasculature and neurons [3]. Sex hormones do not fully account for all sex differences, it has also been demonstrated that neuronal apoptosis pathways differ between males and females [10] and that male neurons are also more sensitive towards nitrosative stress [11]. We have demonstrated that there are differences in the cerebrovascular receptor expression in males versus females both in human brain vessels and in rats after transient middle cerebral artery occlusion (tMCAO, a standard method for this type of experimental stroke) [12, 13].

Ischemic stroke is primarily a vascular disease and we hypothesize that reperfusion and subsequent protection of the brain against hemorrhage, inflammation and edema by targeting the cerebral arteries is the first step towards successful stroke treatment [14]. The BBB consists of endothelial cells with continuous tight junctions, which offer protection against the pathogens, toxins and reduce the influence of the peripheral immune system in the brain. The endothelial cells are supported by the vascular smooth muscle cells (VSMCs), astrocytes, pericytes and the extracellular matrix (ECM). This system is disrupted after stroke due to formation of reactive oxygen species (ROS) and subsequent inflammatory processes [5]. This allows peripheral inflammatory cells to migrate across the BBB and cause further destruction to the brain tissue. In addition to endothelial damage, reperfusion puts a strain on the VSMCs, causing enhanced vasocontractile responses which reduce perfusion [14]. The VSMCs also express inflammatory cytokines in response to ischemia-reperfusion, including metalloproteinases, which contribute to recruitment of inflammatory cells and further BBB breakdown [15]. This has been verified ex vivo in both rodents following experimental stroke [16, 17] and humans [18].

The present study was conducted to examine the early cerebrovascular processes of vascular damage after stroke in females and subsequently examine if sex differences and similarities in these responses occurring in the cerebral vessel wall exist. After a stroke, there is enhanced activation of phosphorylated extracellular signal-regulated kinase 1 and 2 (pERK1/2) in the cerebral vasculature already after a few minutes which reach even higher levels at 3 h [19]. In order to examine which genes were activated in the early stroke stages (at 3 h), we performed whole-transcriptome expression profiling on middle cerebral arteries (MCAs) of female rats after tMCAO-induced ischemia. This was also done to identify activated biological processes and pathways locally in the MCAs which potentially could be targeted for vascular protection after stroke. In addition to the microarray, we validated selected high-expression genes that potentially are involved in reperfusion injury in male and female rats, and compared the differentially expressed genes in MCAs from females (current study) with MCAs from males (previous study, Grell et al. [20]) after tMCAO to contribute to basic knowledge of vascular wall processes in both sexes after stroke.

## Results

### Physiological parameters for both sexes

The body weight was significant lower in females (243 ± 9 g) than males (323 ± 16 g) (*p* < 0. 05), although they were of the same age. During the occlusion and reperfusion, the blood flow over the MCA distribution area was measured with a laser Doppler flowmetry probe [21, 22]. Insertion of the intraluminal filament resulted in a mean reduction of blood flow by 73 ± 10% in females and by 76 ± 9% in males (*p* > 0.05). Withdrawal of the filament after two hours of occlusion resulted in a mean increase of blood flow by 65 ± 13% in females and by 49 ± 17% in males (p > 0.05). This resulted in a blood flow approximate to the level observed prior to the occlusion. Prior to the occlusion, body temperature, blood pressure, blood glucose, pH, p_a_CO_2_ and p_a_O_2_ were measured. These parameters were within the physiological range and did not differ between the sexes (data not shown).

### Whole transcriptome expression profiling in females

**Fig. 1:**
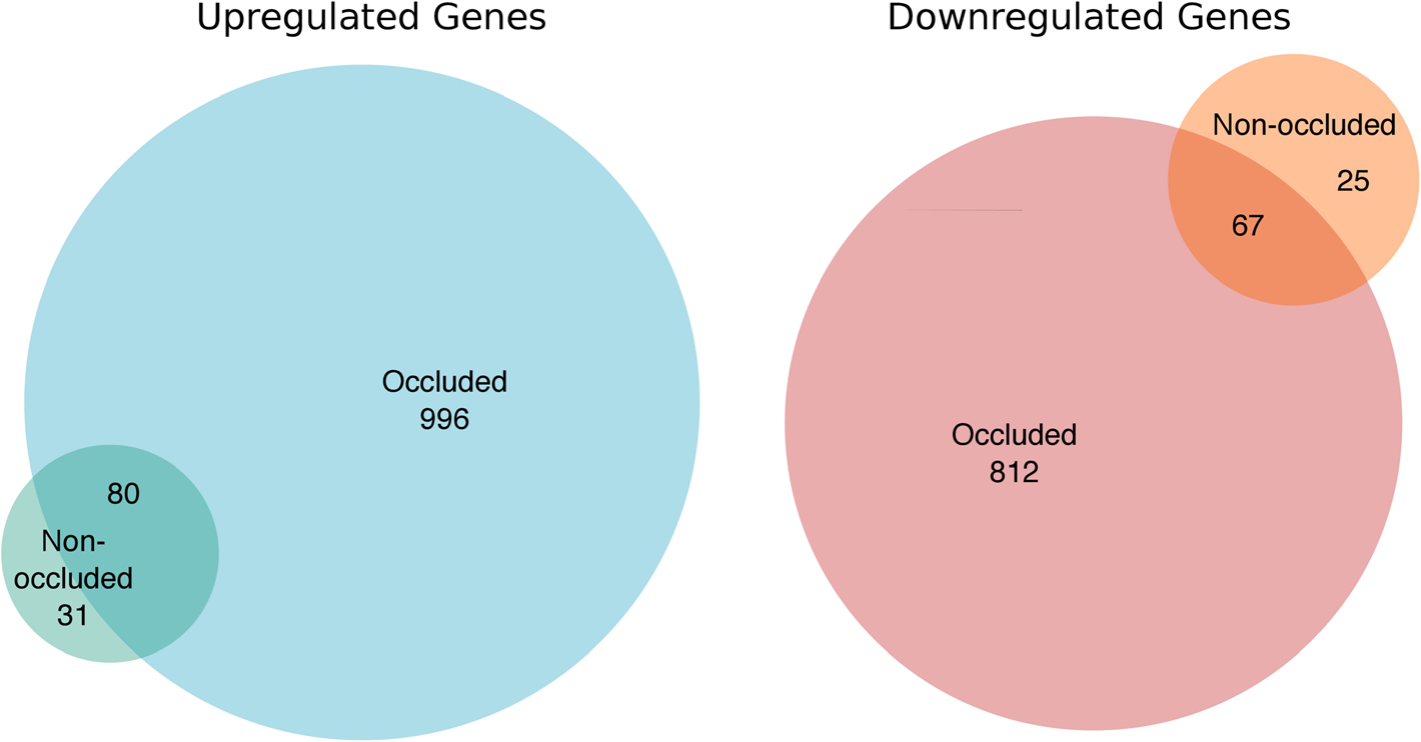
Differentially expressed gene count in the occluded and non-occluded middle cerebral arteries (MCAs) from female rats. Venn diagrams illustrating the number of upregulated and downregulated genes in the occluded and non-occluded MCAs both compared with control MCAs as well as gene overlap between the experimental groups

**Fig. 2:**
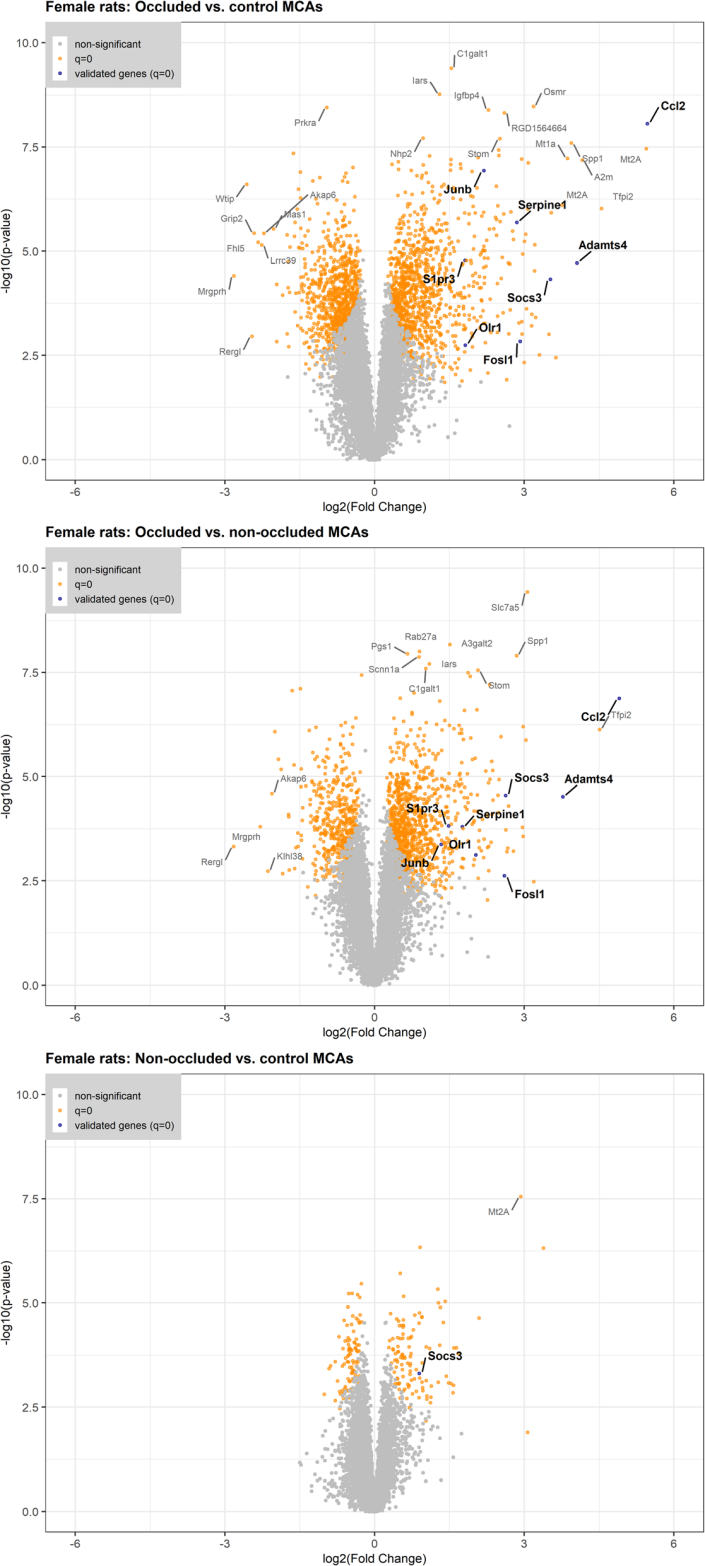
Differentially expressed gene distributions in the occluded and non-occluded middle cerebral arteries (MCAs) from female rats. Volcano plots illustrating distributions of the differentially expressed genes in the occluded and non-occluded MCAs both compared with control MCAs, and in the occluded MCAs compared with the non-occluded MCAs from female rats. Similar Volcano plots for male stroke rats can be found in the publication by Grell et al. [20] (please notice the difference in rat strain and microarray run)

In the microarray analysis, bilateral MCA segments (occluded (n_sample_ = 5) and non-occluded (n_sample_ = 6)) from 6 female rats subjected to tMCAO were analyzed. The arteries were removed 3 h post-reperfusion. MCAs from 6 healthy female rats were used as controls. In total, 1076 genes showed an increased expression and 879 genes had a decreased expression in the occluded MCAs as compared with the control MCAs. In the contralateral non-occluded MCAs, 111 genes had an increased expression and 92 genes had a decreased expression. The expressions of 80 of the differentially expressed genes were increased in both the occluded and non-occluded MCAs while the expressions of 67 of the differentially expressed genes were decreased in both the occluded and non-occluded MCAs (Fig. 1, Fig. 2).

### Gene and protein ontology enrichment analysis in females

#### Occluded MCAs compared with control arteries

**Fig. 3:**
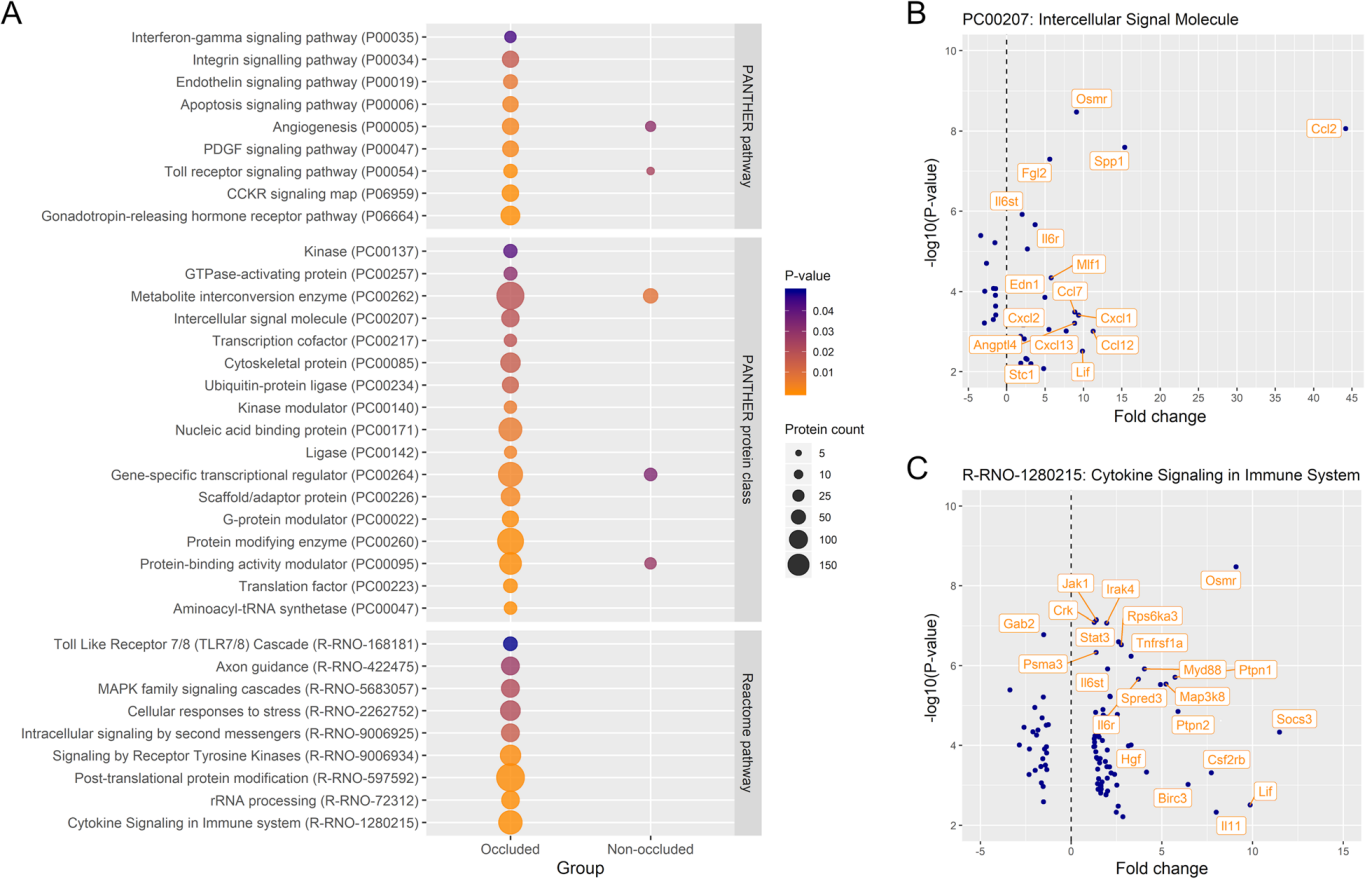
Overrepresented protein classes and pathways in the occluded middle cerebral artery (MCA) from female rats. **a**. Seventeen PANTHER protein classes and 18 PANTHER and Reactome pathways were overrepresented amongst the differentially expressed genes in the occluded MCAs from female rats compared with control MCAs. In the non-occluded MCAs, only 5 overrepresented pathways and protein classes were identified all of which were also identified for the occluded MCAs. **b**. Scatterplot illustrating the differentially expressed genes annotated to the overrepresented PANTHER protein class, intercellular signal molecule, with fold change on the x-axis and -log10(*p*-value) on the y-axis (highlighted if fold change ≥4 or -log10(*p*-value) ≥ 5.5). Chemokine (C-C motif) ligand 2 (*Ccl2*) had the highest fold change within this protein class. **c**. Scatterplot illustrating the differentially expressed genes annotated to the overrepresented Reactome pathway, cytokine signaling in immune system, with fold change on the x-axis and -log10(*p*-value) on the y-axis (highlighted if fold change ≥4 or -log10(*p*-value) ≥ 5.5). Suppressor of cytokine signaling 3 (*Socs3*) had the highest fold change and second lowest *p*-value within this pathway

With the significantly differentially expressed genes in the microarray, a gene ontology (GO) enrichment analysis was performed to identify activated biological processes. When comparing the occluded MCAs to control MCAs, 91 GO terms within the biological process domain were overrepresented (the top findings are presented in Table 1). Examples of other overrepresented GO terms of interest than those presented in Table 1 include: ‘Regulation of epithelial cell migration’ (GO:0010632; annotation of 47 differentially expressed genes), ‘Regulation of neuron projection development’ (GO:0010975; annotation of 91 differentially expressed genes) and ‘Regulation of cytoskeleton organization’ (GO:0051493; annotation of 83 differentially expressed genes). Protein ANalysis THrough Evolutionary Relationships (PANTHER) and Reactome enrichment analyses were also carried out. The genes differentially expressed in the occluded MCAs in relation to control MCAs were overrepresented for 17 PANTHER protein classes, 9 PANTHER pathways and 9 Reactome pathways (Fig. 3a).

**Table 1:** Gene enrichment analysis for the occluded middle cerebral arteries (MCAs) from female rats. Overview of the top 15 overrepresented gene ontology (GO) biological process terms with the highest fold enrichment and top 15 overrepresented GO biological process terms with lowest *p*-value identified for the differentially expressed genes in the occluded MCAs compared with control MCAs

Category	Biological process, GO ID	***P***-value	FE^**a**^	Freq in Geneset	Freq in Genome
Protein synthesis and modification	rRNA processing, GO:0006364	7.20E-09	3.25	58	216
	Protein autophosphorylation, GO:0046777	1.23E-05	2.94	48	198
	Positive regulation of transcription by RNA polymerase II, GO:0045944	1.21E-08	1.77	186	1270
Oxidative stress	Response to hydrogen peroxide, GO:0042542	4.19E-06	3.25	44	164
	Cellular response to reactive oxygen species, GO:0034614	6.48E-04	2.88	39	164
	Cellular response to hypoxia, GO:0071456	1.69E-03	2.86	37	157
	Regulation of reactive oxygen species metabolic process, GO:2000377	2.54E-03	2.54	44	210
Inflammation	Cellular response to interleukin-1, GO:0071347	4.75E-04	3.22	34	128
	Cellular response to tumor necrosis factor, GO:0071356	4.10E-04	2.73	44	195
	Cellular response to lipopolysaccharide, GO:0071222	1.84E-05	2.72	53	236
	Inflammatory response, GO:0006954	2.12E-05	2.22	78	425
Molecular “switches”	Stress-activated protein kinase signaling cascade, GO:0031098	2.61E-03	3.16	31	119
	Response to cAMP, GO:0051591	1.68E-03	2.91	36	150
	Positive regulation of GTPase activity, GO:0043547	1.84E-06	2.49	70	341
	G protein-coupled receptor signaling pathway, GO:0007186	1.64E-21	0.32	55	2088
Tissue repair and recovery	Positive regulation of angiogenesis, GO:0045766	5.50E-04	2.81	42	181
	Positive regulation of cell migration, GO:0030335	8.91E-11	2.38	111	565
	Cellular response to growth factor stimulus, GO:0071363	7.66E-06	2.10	92	531
	Epithelium development, GO:0060429	1.83E-05	1.72	150	1058
Cell death	Intrinsic apoptotic signaling pathway, GO:0097193	3.91E-02	2.63	33	152
	Negative regulation of apoptotic process, GO:0043066	1.76E-05	1.78	137	934
Other	Cellular response to antibiotic, GO:0071236	1.04E-03	2.76	41	180
	Regulation of cellular response to stress, GO:0080135	2.47E-08	2.10	119	688
	Cellular response to organic cyclic compound, GO:0071407	1.21E-06	2.02	110	661
	Negative regulation of multicellular organismal process, GO:0051241	5.19E-12	1.88	204	1312

^a^FE, fold enrichment

**Fig. 4:**
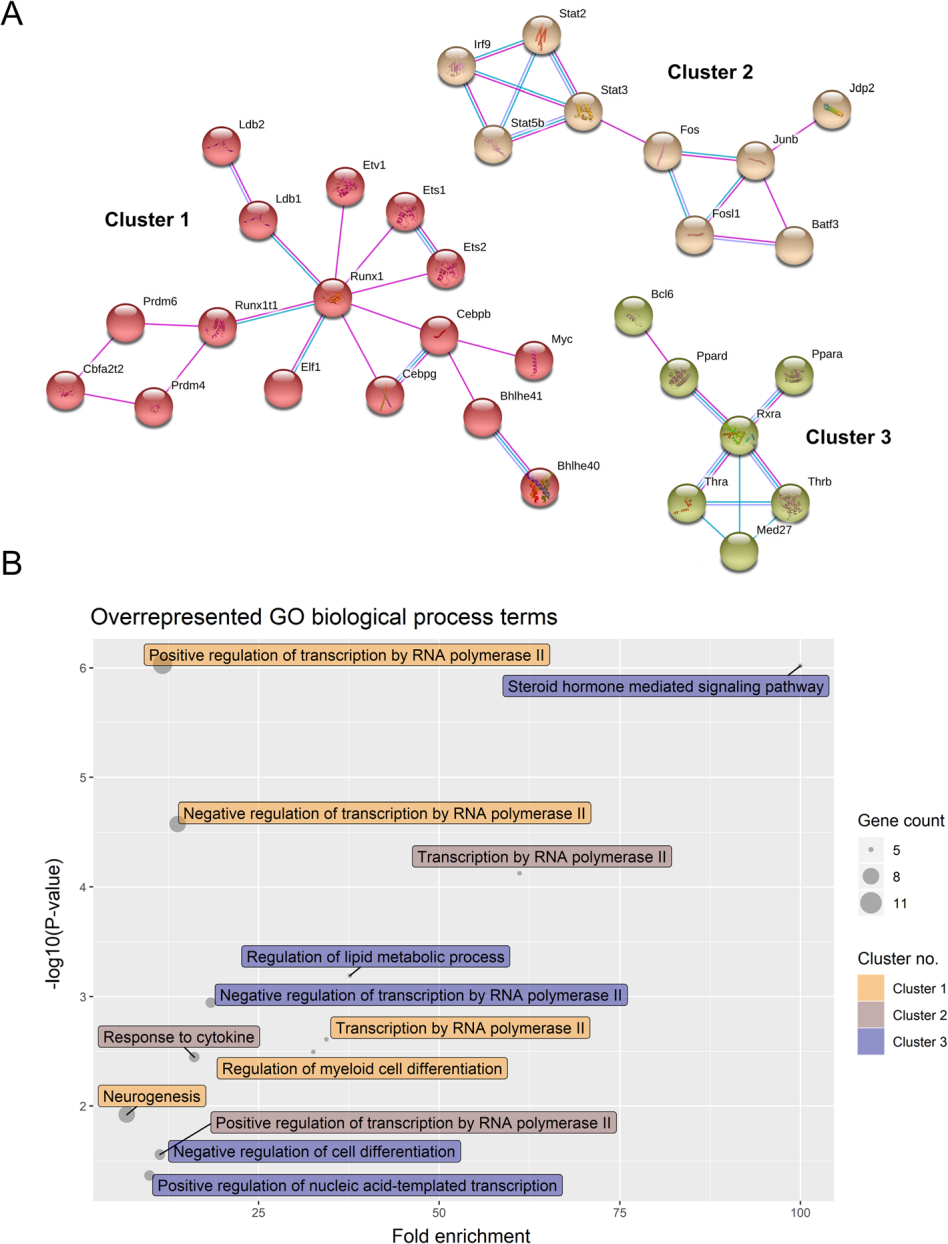
Transcriptional regulators as potential therapeutic targets in ischemic stroke treatment for female animals. **a**. Medium-confidence STRING network showing *Rattus norvegicus* protein-protein interactions of differentially expressed gene products annotated to the PANTHER protein class, gene-specific transcriptional regulator (108 annotated genes from the geneset), for the occluded middle cerebral artery experimental group. We identified 3 clusters formed by 16, 9 and 7 gene products. **b**. Five, 3 and 5 overrepresented GO biological process terms (cut-off: at least 5 annotated genes) were identified for cluster 1, 2 and 3 shown in **a**, respectively. The gene-specific transcriptional regulators were involved in biological processes such as neurogenesis, response to cytokine and regulation of cell differentiation

**Fig. 5:**
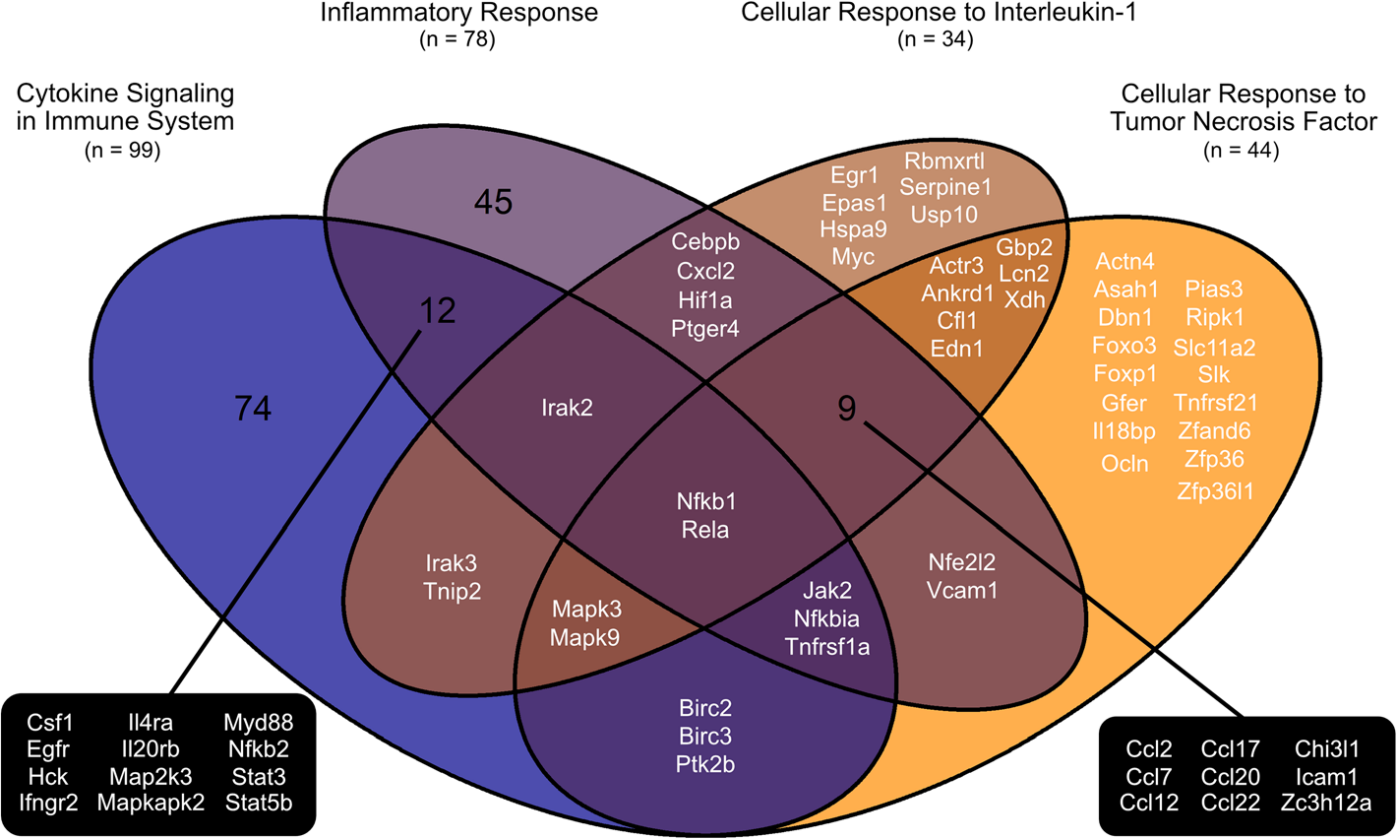
Inflammation as potential therapeutic targets in ischemic stroke treatment for female animals. Venn diagram visualizing differentially expressed genes in the occluded middle cerebral artery compared with control MCAs that overlaps between the overrepresented Reactome pathway, cytokine signaling in immune system (Fig. 3), and overrepresented GO biological process terms, inflammatory response, cellular response to interleukin-1 and cellular response to tumor necrosis factor (Table 1). Nuclear factor of kappa light polypeptide gene enhancer in B-cells 1 (*Nfkb1*) and nuclear factor kappaB subunit p65 (*Rela*) were annotated to each of them. Focusing solely on the GO biological process terms, this was also the case for intercellular adhesion molecule 1 (*Icam1*), zinc finger CCCH type containing 12A (*Zc3h12a*), chitinase-3-like protein 1 (*Chi3l1*) and several chemokines (e.g. C-C motif chemokine 2 (*Ccl2*)), in addition to *Nfkb1* and *Rela*

Across analyses, an enrichment of genes related to apoptosis, regulation of transcription, protein autophosphorylation, inflammation, oxidative stress, and tissue repair and recovery could be seen (Table 1 and Figs. 3, 4 and 5). Within the overrepresented PANTHER protein class ‘Intercellular signal molecule’ (PC00207), chemokine (C-C motif) ligand 2 (*Ccl2*) had the highest fold change (Fig. 3b). *Ccl2* is also annotated to the overrepresented GO terms ‘Cellular response to interleukin-1’ (GO:0071347) and ‘Cellular response to tumor necrosis factor’ (GO:0071356) amongst other cytokines (Fig. 5). Within the overrepresented Reactome pathway ‘Cytokine signaling in immune system’ (R-RNO-1280215), suppressor of cytokine signaling 3 (*Socs3*) had the highest fold change (Fig. 3c). This was also the case amongst the kinase modulators (Table 2).

**Table 2:** Gene-specific transcriptional regulators and kinase modulators in the occluded middle cerebral arteries (MCAs) from female rats. Overview of the differentially expressed genes, annotated to a subontology of the overrepresented gene-specific transcriptional regulator (PC00264) and kinase modulator (PC00140) PANTHER protein classes, in the occluded MCAs compared with control MCAs

Gene symbol	Gene description	***P***-value	Fold change
**PC00056: Basic Leucine Zipper Transcription Factor**			
*Fosl1*	Fos-like antigen 1	0.002	7.55
*JunB*	Jun B proto-oncogene	1.17E-07	4.56
*Fos*	FBJ osteosarcoma oncogene	0.006	2.85
*Maff*	V-maf musculoaponeurotic fibrosarcoma oncogene homolog F	6.65E-05	2.38
*Cebpb*	CCAAT/enhancer binding protein (C/EBP), beta	8.87E-05	2.08
*Mafk*	V-maf musculoaponeurotic fibrosarcoma oncogene homolog K	3.20E-05	1.79
*Cebpg*	CCAAT/enhancer binding protein (C/EBP), gamma	4.94E-05	1.71
*Nfe2l2*	Nuclear factor, erythroid derived 2, like 2	5.92E-05	1.55
*Jdp2*	Jun dimerization protein 2	1.26E-04	1.37
*Batf3*	Basic leucine zipper transcription factor, ATF-like 3	2.04E-04	1.31
**PC00140: Kinase Modulator**			
*Socs3*	Suppressor of cytokine signaling 3	4.68E-05	11.48
*Cish*	Cytokine inducible SH2-containing protein	6.03E-06	2.23
*Socs2*	Suppressor of cytokine signaling 2	0.002	2.22
*Ccnl1*	Cyclin L1	5.06E-04	2.05
*Pik3r1*	Phosphoinositide-3-kinase, regulatory subunit 1 (alpha)	1.97E-05	1.98
*Mob3a*	MOB kinase activator 3A	3.38E-04	1.91
*Ccnh*	Cyclin H	1.11E-04	1.50
*Pkig*	Protein kinase inhibitor, gamma	2.64E-04	1.45
*Socs5*	Suppressor of cytokine signaling 5	1.49E-05	1.35
*Prkag1*	Protein kinase, AMP-activated, gamma 1 non-catalytic subunit	1.05E-04	−1.28
*Phka1*	Phosphorylase kinase, alpha 1	8.80E-04	−1.52
*Pik3r2*	Phosphoinositide-3-kinase, regulatory subunit 2 (beta)	2.05E-05	−1.60
*Mob2*	MOB kinase activator 2	7.56E-04	−2.48

#### Occluded and non-occluded MCAs compared with control arteries

The number of differentially expressed genes after experimental stroke was considerable higher in the occluded MCAs (1955 differentially expressed genes) than in the non-occluded MCAs where only 203 differentially expressed genes were identified (Fig. 1). To reveal if the biological processes activated in the occluded MCAs are similar to those activated in the non-occluded MCAs, we looked for overlapping overrepresented GO terms. When using the predefined selection criteria, no overlap was observed. When no predefined selection criteria were applied, overlap was observed between the two experimental groups. A selection of overlapping overrepresented GO terms can be found in Table 3 (relaxed criteria). In addition to the GO enrichment analysis, 2 PANTHER pathways (‘Angiogenesis’ (P00005) and ‘Toll receptor signaling pathway’ (P00054)) and 3 PANTHER protein classes (‘Protein-binding activity modulator’ (PC00095), ‘Metabolite interconversion enzyme’ (PC00262) and ‘Gene-specific transcriptional regulator’ (PC00264)) were overrepresented in the list of differentially expressed genes for both the occluded and non-occluded MCAs when compared with control MCAs (Fig. 3a).

**Table 3:** Gene enrichment analysis for the occluded and non-occluded middle cerebral arteries (MCAs) from female rats. Selected overlapping overrepresented biological process gene ontology (GO) terms identified for both the occluded and non-occluded MCAs when compared with control MCAs

Biological process, GO ID	Occluded MCAs			Non-occluded MCAs		
	Freq in Geneset	FE^a^	***P***-value	Freq in Geneset	FE^a^	***P***-value
Response to oxygen-containing compound, GO:1901700	315	1.9	5.06E-22	49	2.8	4.39E-07
Regulation of cell communication, GO:0010646	455	1.7	1.52E-21	61	2.1	1.61E-04
Response to stress, GO:0006950	445	1.7	3.11E-21	63	2.2	7.74E-06
Regulation of gene expression, GO:0010468	507	1.6	3.98E-20	64	1.8	4.13E-03
Response to cytokine, GO:0034097	171	2.3	5.38E-17	24	3.0	0.02
Response to lipid, GO:0033993	193	1.9	2.37E-12	35	3.3	7.56E-06
Regulation of transcription by RNA polymerase II, GO:0006357	281	1.7	4.94E-12	44	2.5	2.78E-04
Regulation of transcription, DNA-templated, GO:0006355	374	1.5	4.92E-11	56	2.1	4.04E-04
Tissue development, GO:0009888	245	1.7	1.25E-10	40	2.6	2.61E-04
Positive regulation of intracellular signal transduction, GO:1902533	153	1.9	3.26E-08	25	2.9	0.03
Transmembrane receptor protein tyrosine kinase signaling pathway, GO:0007169	69	2.4	9.91E-06	14	4.5	0.04

^a^FE: Fold enrichment

Focusing on the gene-specific transcriptional regulators, 108 and 15 differentially expressed genes in the occluded and non-occluded MCAs were annotated to this GO term, respectively. Of these, twelve differentially expressed genes were found for both experimental groups (e.g. PR domain zinc finger protein 4 (*Prdm4*), Runt-related transcription factor 1 (*Runx1*) and signal transducer and activator of transcription 3 (*Stat3*)). Focusing on the occluded MCAs, 94 of the gene-specific transcriptional regulators were DNA-binding transcription factors (PC00218). Ten of those can more specifically be categorized as basic leucine zipper transcription factors (PC00056, Table 2). For the 108 gene-specific transcriptional regulators, we identified 3 protein-protein interaction clusters formed by 32 of these regulators (e.g. *Runx1*, Fos-like antigen 1 (*Fosl1*) and Jun B proto-oncogene (*JunB*); Fig. 4a). For each cluster, we identified overrepresented GO terms within the biological process domain. The regulators forming cluster 2 (*Fosl1* and *JunB* is a part of this cluster) are involved in transcription and cytokine response (Fig. 4b).

### qPCR for validation of target genes for both sexes

**Fig. 6:**
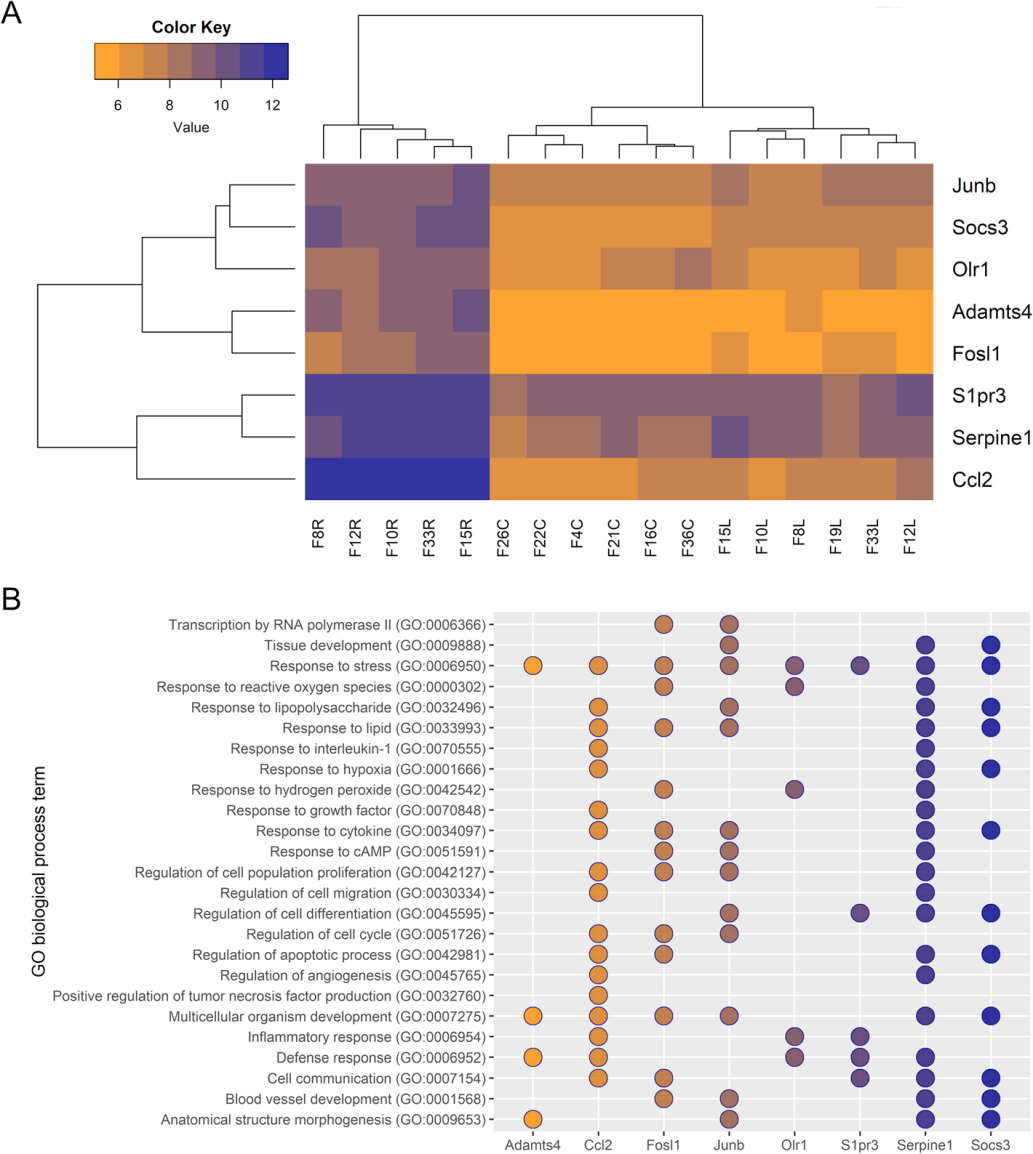
Further analysis of the upregulated gene targets in the female rat middle cerebral arteries (MCAs). **a**. Heatmap and dendrograms illustrating hierarchical clustering of the expression of the gene targets for the experimental groups, occluded MCAs (R), non-occluded MCAs (L) and control MCAs (C), based on outcomes from the Affymetrix whole-transcriptome expression profiling (microarray analysis). The highest gene expression of ADAM metallopeptidase with thrombospondin type 1 motif 4 (*Adamts4*), C-C motif chemokine 2 (*Ccl2*), Fos-like antigen 1 (*Fosl1*), Jun B proto-oncogene (*JunB*), oxidized low density lipoprotein receptor 1 (*Olr1*), sphingosine-1-phosphate receptor 3 (*S1pr3*), serpin peptidase inhibitor, clade E member 1 (*Serpine1*) and suppressor of cytokine signaling 3 (*Socs3*) were observed for the occluded MCAs. **b**. Dot plot showing categorization of the gene targets, *Adamts4*, *Ccl2*, *Fosl1*, *JunB*, *Olr1*, *S1pr3*, *Serpine1* and *Socs3*, into selected GO terms within the biological process domain. All gene targets were annotated to response to stress

To validate the results from the microarray, eight high-expression genes of interest were chosen for quantitative real-time polymerase chain reaction (qPCR) analysis in MCAs from both female and male rats (a new set of animals were operated on in both sexes, 5 stroke females and 6 stroke males were included in the analysis as well 6 controls of each sex). The expression of the target genes in the occluded MCAs, non-occluded MCAs and control MCAs from the microarray analysis can be found in Fig. 2 and Fig. 6a. To get an increased understanding of what biological processes the target genes are involved in, we categorized them into preselected GO terms. The target genes are all involved in response to stress. Other GO terms of interest include cell communication, defense response and response to cytokine (Fig. 6b).

**Fig. 7:**
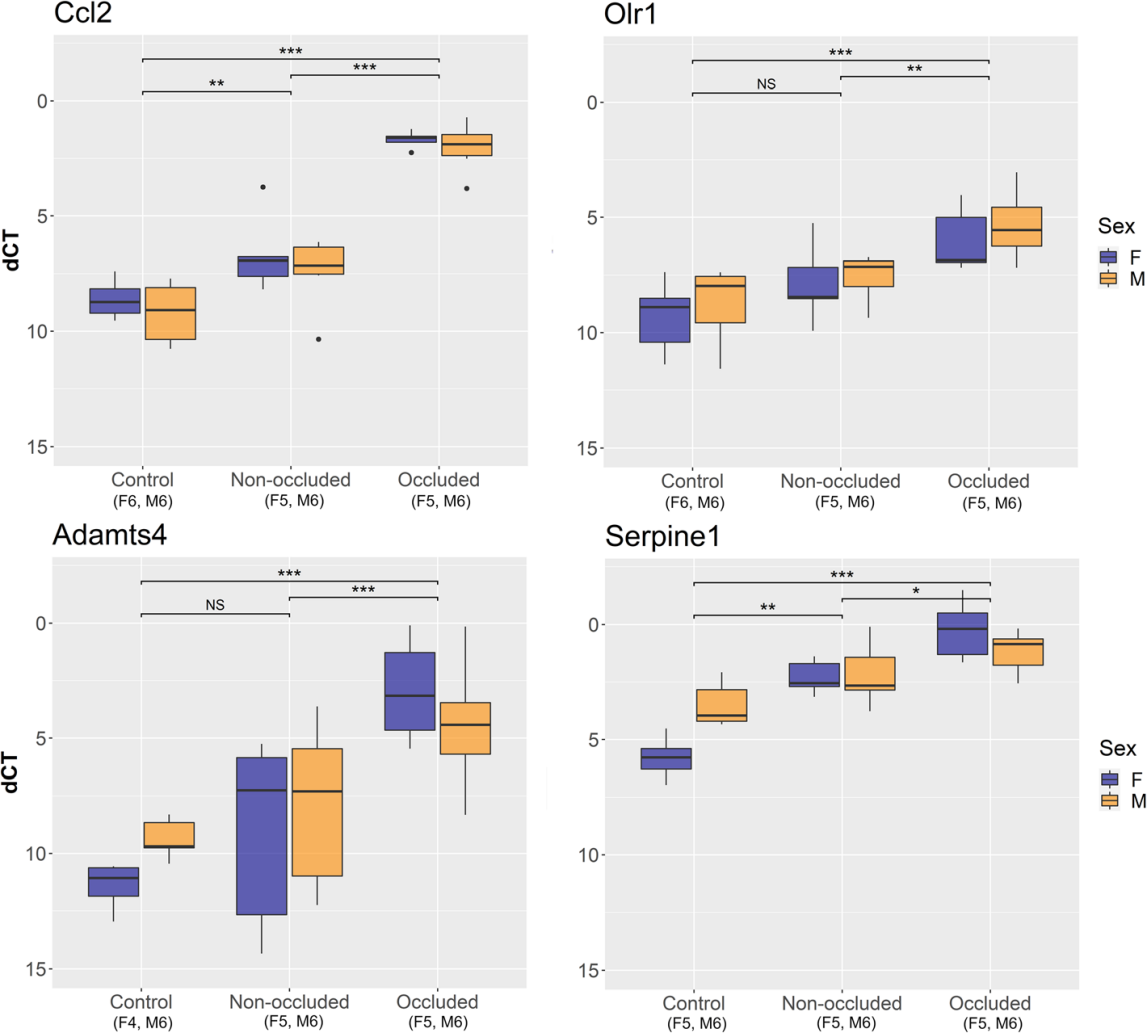
Validation of gene targets in male and female middle cerebral arteries (MCAs) after tMCAO. Differential gene expression of the selected upregulated gene targets was validated by qPCR. When comparing delta cycle threshold (dCT) values between the (non-operated) control MCAs, and the non-occluded and occluded MCAs after tMCAO [transient middle cerebral artery occlusion], significant elevations were seen in both sexes for C-C motif chemokine 2 (*Ccl2*), oxidized low-density lipoprotein receptor 1 (*Olr1*), a disintegrin and metalloproteinase with thrombospondin type 1 motif, 4 (*Adamts4*), and serine protease inhibitor, clade E, member 1 (*Serpine1*, one outlier excluded for this gene dataset). Sex did not significantly affect the outcome for any of the genes being validated (*p* = 0.22–0.52) therefore, only the fixed effect 'experimental group' was fitted into the model (*p* < 0.01). First, we compared the occluded and non-occluded MCAs to the reference ‘control MCAs’, and second, we compared the occluded MCAs to the reference ‘non-occluded MCAs’. Information within parentheses shows number of samples for the sex (F/M) and group in question. Statistical tests were performed (see methods): * indicates *p* < 0.05, ** indicates p < 0.01, *** indicates *p* < 0.001 and NS indicates non-significance (*p* > 0.05). *Abbreviations: F, female; M, Male*

**Fig. 8:**
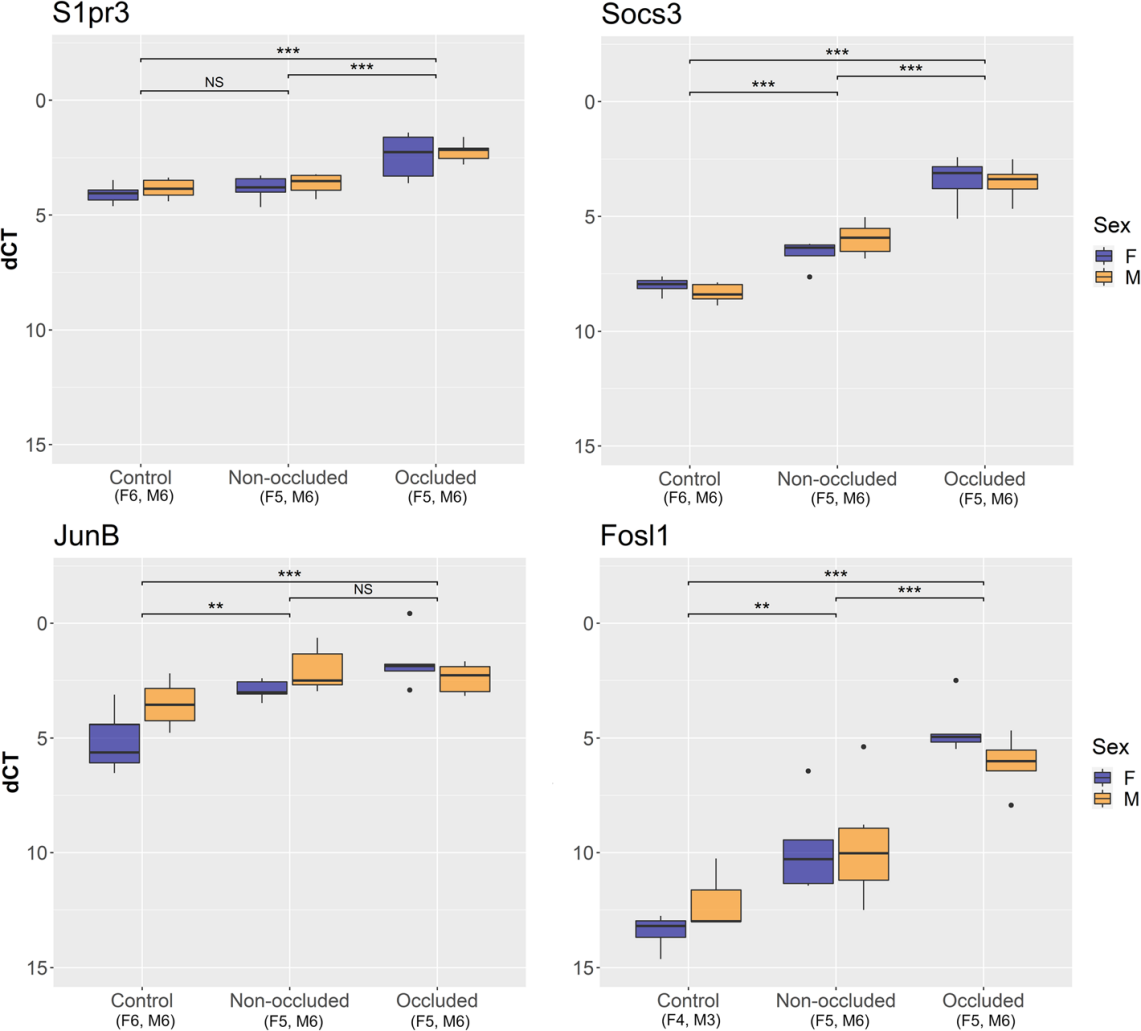
Validation of gene targets in male and female middle cerebral arteries (MCAs) after tMCAO (continued). Differential gene expression of the selected upregulated gene targets was validated by qPCR. When comparing delta cycle threshold (dCT) values between the (non-operated) control MCAs, and the non-occluded and occluded MCAs after tMCAO [transient middle cerebral artery occlusion], significant elevations were seen in both sexes for sphingosine 1 phosphate receptor 3 (*S1pr3*), suppressor of cytokine signaling 3 (*Socs3*), Jun B proto-oncogene (*JunB*), and Fos-like antigen 1 (*Fosl1*). Sex did not significantly affect the outcome for any of the genes being validated (*p* = 0.11–0.87) therefore, only the fixed effect 'experimental group' was fitted into the model (*p* < 0.01). First, we compared the occluded and non-occluded MCAs to the reference ‘control MCAs’, and second, we compared the occluded MCAs to the reference ‘non-occluded MCAs’. Information within parentheses shows number of samples for the sex (F/M) and group in question. Statistical tests were performed (see methods): * indicates *p* < 0.05, ** indicates *p* < 0.01, *** indicates *p* < 0.001 and NS indicates non-significance (*p* > 0.05). *Abbreviations: F, female; M, Male*

For qPCR, glyceraldehyde 3-phosphate dehydrogenase (*Gapdh*) and actin B (*ActB*) were used as reference genes; an equal stable high expression was confirmed throughout the groups (data not shown). All of the 8 analyzed genes (*Ccl2*, oxidized low-density lipoprotein receptor 1 (*Olr1*), a disintegrin and metalloproteinase with thrombospondin type 1 motif, 4 (*Adamts4*), serine protease inhibitor, clade E, member 1 (*Serpine1*), sphingosine 1 phosphate receptor 3 (*S1pr3*), *Socs3*, *JunB* and *Fosl1*) were significantly upregulated in the occluded MCAs compared with control MCAs (Figs. 7 and 8). In addition, *Ccl2, Socs3*, *Fosl1*, *JunB* and *Serpine1* were also upregulated in the non-occluded MCAs as compared to control MCAs. Sex did not have a significant effect on the expression of any of the 8 analyzed genes (*p* = 0.11–0.87, Figs. 7 and 8).

### Cross-analysis to reveal sex similarities

**Fig. 9:**
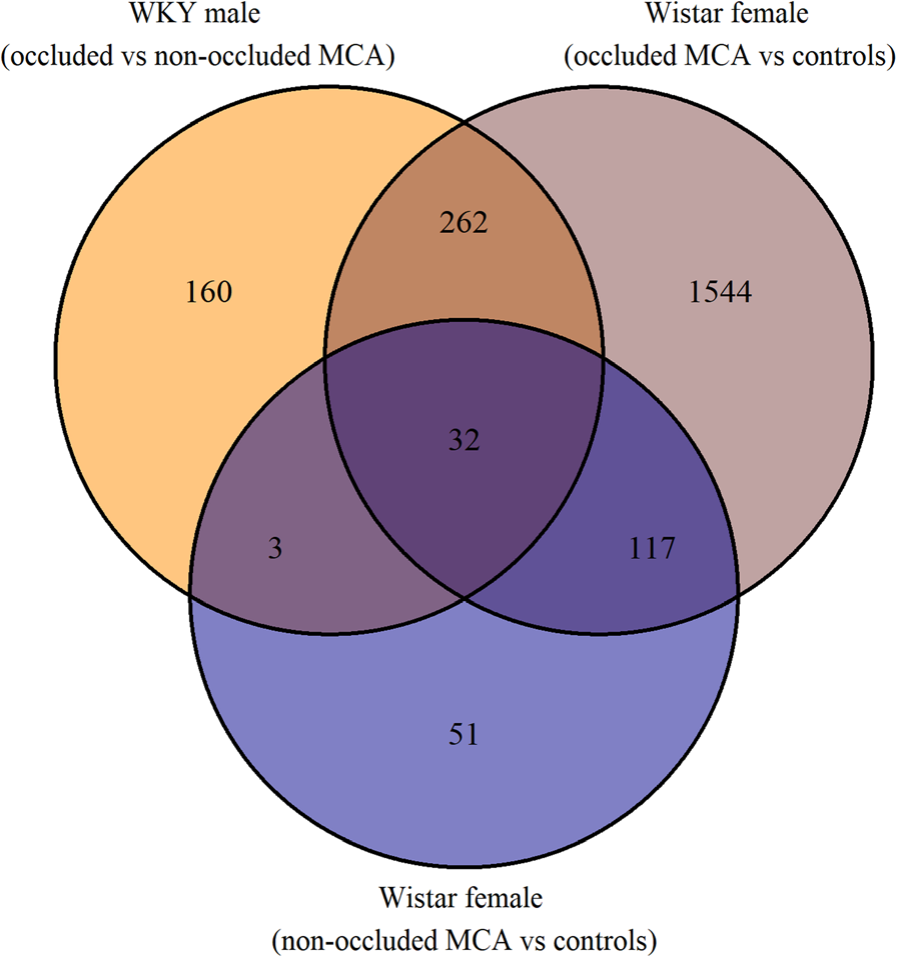
Overlapping differentially expressed genes between three experimental tMCAO stroke groups (current and previous study). Venn diagram illustrating the number of overlapping differentially expressed genes between the following experimental stroke groups (tMCAO [transient middle cerebral artery occlusion] stroke model): (i) Occluded versus non-occluded middle cerebral arteries (MCAs) from Wistar-Kyoto (WKY) male rats (results from Grell et al. [20]), (ii) Occluded MCAs vs control MCAs from Wistar female rats, and (iii) Non-occluded MCAs vs control MCAs from Wistar female rats. Here, we identified an overlap of 149 genes between group (ii) and (iii) in contrast to 147 genes observed in Fig. 1 where both upregulation and downregulation of the genes were considered. The reason for the discrepancy is due to (1) Tetratricopeptide repeat domain 39B (*Ttc39b*) is downregulated in the non-occluded MCAs and upregulated in the occluded MCAs when compared with control MCAs, and (2) FK506 binding protein 3 (*Fkbp3*) is upregulated in the non-occluded MCAs and downregulated in the occluded MCAs when compared with control MCAs

The comparison with findings presented by Grell et al. [20] revealed overlap between the differential expressed genes identified in the occluded versus non-occluded MCAs from Wistar Kyoto (WKY) male rats, the occluded MCAs versus control MCAs from Wistar female rats and the non-occluded MCAs versus control MCAs from Wistar female rats. Thirty-two genes were differentially expressed in each of the experimental groups (Fig. 9). An overview of those genes is presented in Table 4.

**Table 4:** Overlapping differentially expressed genes found for each of the experimental tMCAO stroke groups (Fig. 9). Thirty-two differentially expressed genes found for each of the following experimental stroke groups (tMCAO [transient middle cerebral artery occlusion] stroke models): Occluded versus non-occluded middle cerebral arteries (MCAs) from Wistar-Kyoto (WKY) male rats (results from Grell et al. [20]), (ii) occluded MCAs vs control MCAs from Wistar female rats, and (iii) non-occluded MCAs vs control MCAs from Wistar female rats. The number of overlapping genes can be found in Fig. 9

Gene symbol	Gene description	WKY male rats (occluded vs non-occluded MCAs)		Wistar female rats (occluded MCAs vs control MCAs)		Wistar female rats (non-occluded MCAs vs control MCAs)	
		***P***-value	Fold change	***P***-value	Fold change	***P***-value	Fold change
*A2m*	Alpha-2-macroglobulin	1.95E-05	4.99	6.50E-08	17.90	1.83E-03	2.19
*Acsl1*	Acyl-CoA synthetase long-chain family member 1	1.94E-04	−1.68	8.00E-04	−2.40	1.23E-03	− 1.42
*Adamts9*	A disintegrin-like and metalloprotease (reprolysin type) with thrombospondin type 1 motif, 9	3.38E-04	2.29	1.17E-05	7.83	5.14E-04	1.64
*Asns*	Asparagine synthetase	1.44E-05	2.52	3.93E-06	8.16	1.92E-04	1.64
*Bcl3*	B-cell CLL/lymphoma 3	6.42E-03	3.03	5.86E-06	8.01	2.17E-05	1.92
*Bri3*	Brain protein I3	1.25E-04	−1.58	1.66E-04	−2.56	6.95E-04	− 1.29
*Cd200*	Cd200 molecule	2.68E-03	1.80	8.15E-08	3.30	1.72E-05	1.86
*Ifitm3*	Interferon induced transmembrane protein 3	2.43E-05	2.02	2.18E-07	2.98	3.77E-04	1.48
*Itga5*	Integrin, alpha 5 (fibronectin receptor, alpha polypeptide)	1.57E-03	2.03	3.71E-04	4.37	1.13E-04	1.61
*Lcn2*	Lipocalin 2	6.31E-04	3.47	3.60E-03	12.39	1.28E-02	8.36
*Litaf*	Lipopolysaccharide-induced TNF factor	3.07E-04	2.32	3.30E-06	6.81	4.53E-04	1.32
*Manba*	Mannosidase, beta A, lysosomal	8.27E-05	−1.52	8.37E-05	−2.03	7.56E-04	−1.41
*Map 3 k6*	Mitogen-activated protein kinase kinase kinase 6	4.36E-04	1.86	1.45E-06	4.43	1.00E-05	2.43
*Mt1a*	Metallothionein 1a	3.02E-03	2.40	6.04E-08	11.14	1.11E-04	2.79
*Mt2A*	Metallothionein 2A	3.61E-03	3.05	4.14E-07	28.56	2.56E-07	9.03
*Mtss1l*	Metastasis suppressor 1-like	2.13E-03	−1.68	3.25E-07	−2.88	1.86E-03	−1.43
*Nampt*	Nicotinamide phosphoribosyltransferase	3.10E-04	1.65	2.82E-07	2.54	4.63E-07	1.88
*Osmr*	Oncostatin M receptor	1.07E-03	2.55	3.35E-09	9.09	4.70E-06	2.41
*Pfkm*	Phosphofructokinase, muscle	1.70E-04	−1.67	4.68E-05	−2.16	1.37E-04	−1.29
*Pla1a*	Phospholipase A1 member A	1.21E-05	2.58	1.48E-04	4.41	1.87E-03	1.85
*Plxdc2*	Plexin domain containing 2	1.40E-04	−1.43	2.02E-05	−1.95	2.04E-05	−1.35
*Ptpn2*	Protein tyrosine phosphatase, non-receptor type 2	2.89E-03	2.03	1.41E-05	5.89	6.99E-05	1.48
*RGD1564664*	Similar to LOC387763 protein	6.82E-04	2.59	4.79E-09	6.07	1.64E-04	1.66
*Runx1*	Runt-related transcription factor 1	1.24E-03	2.60	1.46E-05	4.59	1.93E-05	1.75
*Sbno2*	Strawberry notch homolog 2 (Drosophila)	3.03E-03	2.45	5.29E-06	6.87	2.90E-05	2.60
*Slc29a2*	Solute carrier family 29 (nucleoside transporters), member 2	1.74E-04	2.17	2.29E-04	2.85	6.60E-04	1.38
*Sod2*	Superoxide dismutase 2, mitochondrial	3.33E-06	2.72	9.45E-06	4.50	3.26E-03	1.62
*Spp1*	Secreted phosphoprotein 1	4.45E-04	4.82	2.54E-08	15.39	1.22E-04	2.14
*Tgfbi*	Transforming growth factor, beta induced	2.36E-05	−1.55	2.37E-04	−1.54	7.57E-04	−1.42
*Tmem51*	Transmembrane protein 51	3.39E-04	−1.65	1.26E-07	−2.80	1.26E-04	−1.42
*Tnfrsf1a*	Tumor necrosis factor receptor superfamily, member 1a	3.12E-04	1.73	5.77E-07	3.30	2.53E-05	1.49
*Uap1*	UDP-N-acteylglucosamine pyrophosphorylase 1	1.29E-03	1.72	4.24E-05	4.60	9.51E-04	1.61

## Discussion

This is to our knowledge the first study to explore possible mechanisms of reperfusion injury after stroke in females by performing a whole transcriptome microarray on isolated MCAs 3 h after the end of a 2-h tMCAO. This unique approach has allowed us to identify potential targets for treatment of vascular injury associated with stroke. The tMCAO model was used to mimic stroke conditions and the cerebral arteries were examined at an early time point to explore the events that occur in VSMCs and endothelial cells that lead to BBB breakdown and endothelial dysfunction. At a similar early time point, we have demonstrated that there is increased expression of phosphorylated protein kinase C-δ (pPKCδ) and pERK1/2 but not of phosphorylated p38, c-jun N-terminal kinase (pJNK) and pPKCα [19]. This has been verified by protein studies [23] and by specific blockade, using inhibitors of the MEK-ERK pathway which resulted in reduced brain damage [14]. Within this study, whole transcriptome expression profiling revealed a considerable number of differentially expressed genes (more than 2000) in the MCAs from female rats after tMCAO. Interestingly, almost 300 of these genes were also differentially expressed in MCAs from male rats after tMCAO.

The present study was also designed to examine if sex differences affect early gene expression patterns of stroke-associated genes. From the microarray analysis, we found a markedly increased expression of *Adamts4*, *Socs3*, *Fosl1*, *JunB*, *Serpine1*, *Olr1*, *Ccl2* and *S1pr3* in the ischemic artery compared to non-ischemic artery at 3-h post reperfusion in female rats. In a further validation of the genes we compared both sexes and found a similar degree of increase in expression. Thus, the results of this part of the study confirmed an increased expression of genes in the vasculature that contributes to inflammation, endothelial dysfunction and breakdown of the BBB.

*Adamts4* is of interest since it for the first time is shown to be upregulated in the cerebral arteries in response to stroke. *Adamts4* is a metalloproteinase that has been shown to have a regulatory function in inflammation and an ability to inhibit angiogenesis [24, 25]. Other metalloproteinases were also found to be overexpressed in the microarray analysis of the occluded MCA (matrix metallopeptidase 19 (*Mmp19*), *Adamts1*, *Adamts5*, *Adamts9*, ADAM metallopeptidase domain 8 (*Adam8*) and *Adam9*), but not to the same degree as *Adamts4*. *Adamts4* has been shown to promote neurite-outgrowth through the MEK pathway, independent of its proteolytic capacities [26]. It is highly expressed in VSMCs in macrophage-rich areas in the thoracic aorta of patients with thoracic aortic dissection and increased serum levels have been associated with carotid atherosclerotic plaque vulnerability [27, 28]. Based on knowledge about the proteolytic capacities of *Adamts4* from earlier studies, one could hypothesize that *Adamts4* and other metalloproteinases contribute to breakdown of the supporting ECM of the arteries, negatively affecting the integrity of the cerebral arteries, increasing the risk of hemorrhagic transformation and edema [29]. Several of the metalloproteinases and ECM genes were upregulated at 6 and 24 h after subarachnoid hemorrhage (SAH) [17], indicating a process that is ongoing. The time course of the *Mmp8*, *Mmp9* and *Mmp13* were carefully monitored in the MCAs immediately after the SAH up until 48 h in male rats [16].

*Olr1* and *Ccl2* expression have earlier been shown to be upregulated in cerebral arteries following tMCAO in both normotensive and hypertensive male rats [20]. Here, we report that this occurs equally well in male and female MCAs after experimental stroke. The *Olr1* gene codes for a scavenger receptor located on endothelial cells, VSMCs and macrophages and are one of the key molecules in atherogenesis. Oxidized low-density lipoprotein activates the receptor, leading to recruitment of inflammatory cells by increasing chemokine and adhesion molecule levels. Although this process has been well characterized in the settings of developing atherosclerosis, it has not yet been established what role *Olr1* plays in the acute phase after ischemia-reperfusion and in particular in the affected MCAs.

*Ccl2* is a chemokine which recruits monocytes and basophils. Monocytes in turn, secrete further inflammatory cytokines, aggravating the damage further. Clot formation, especially fibrin, triggers activation of monocytes [30] and endothelial cells [31]. Previous studies have shown that the expression of *Ccl2* is highly increased in the brain after stroke, and that silencing the *Ccl2* gene is protective in stroke models [32]. Here we show that the expression is highly elevated in both sexes early after reperfusion which formerly has been seen in hypertensive males 6 h post reperfusion but never shown to be equally increased at this early time point after a tMCAO or in both sexes. We have previously reported on the increased presence of the chemokine (C-X-C motif) ligand 2 (*Cxcl2*) and interleukin-6 (*Il-6*) at gene level in experimental SAH at 24 h [18], and followed the time course from 0 to 48 h of several other cytokines such as tumor necrosis factor α (*TNFα*) and *Il-1β*, and chemokine ligands *Cxcl1* and *Ccl20*, in addition to *Il-6* and *Cxcl2* [16]. Again, this clearly shows that the process is ongoing in the cerebral vessel walls at least 48 h after experimental SAH.

Another gene of interest is *S1pr3* which has an increased expression after ischemia-reperfusion in cerebral arteries. Sphingosine 1-phosphate (S1P) signaling via S1pr3 has been shown to have both protective effects on endothelial function in ischemia/reperfusion in the heart [33] as well as vasocontractile properties when located on the VSMCs in cerebral arteries [34]. Activation of S1pr3 has also been shown to negatively influence endothelial barrier function [35].

Transcription factors and intracellular signaling pathways are most likely more effective treatment targets when it comes to inflammation as opposed to blocking individual specific cytokines or chemokines. Inflammation is crucial for repair and recovery after stroke, but in the initial phase, reduction of inflammation has been shown to be beneficial. By targeting the arteries, we believe that we can reduce the initial recruitment and migration of inflammatory cells. Our data showed that the selected inflammatory regulatory genes *Socs3*, *JunB* and *Fosl1* were overexpressed in both the occluded and non-occluded artery compared to healthy controls, indicating that inflammation is not exclusive to the occluded artery. JunB and Fosl1 are part of the activator protein complex, which make up the transcription factor activator protein 1 (AP-1). AP-1 has been suggested to be responsible for activating inflammation and atherogenesis in response to changes in sheer stress, which could explain why these genes here are shown to be upregulated early after reperfusion [36]. The upstream activator of the AP-1 complex is the stress-activated protein kinases/Jun amino-terminal kinases (SAPK/JNK), which has earlier been shown to be activated in cerebral VSMCs 24 h after SAH, tMCAO and ex vivo organ culture of cerebral arteries [17]. In experimental SAH, we have additionally observed the dynamic activation of two other transcription factors, activating transcription factor–2 (ATF-2) and ETS transcription factor (ELK-1), during the first two days after the induction of the hemorrhage in male rats [16].

### Limitations

The current study focuses solely on the early cerebrovascular processes of vascular damage after a tMCAO-induced stroke. Investigation of long-term effects after a tMCAO-induced stroke, as well as stroke induced by other experimental procedures, would be beneficial on the path to discover new therapeutic targets and to improve our understanding of the stroke pathophysiology.

## Conclusions

This study may pave the way towards novel ways to modify cerebrovascular gene regulation and provide novel therapeutic targets to improve cerebrovascular disorders like stroke. Our findings revealed that following tMCAO there are dynamic alterations in gene expression in the immediate MCA, but also on the contralateral side, albeit to a less pronounced degree. Focusing on the high expression gene targets, we observed no significant sex-dependent difference (pattern of changes in males was similar to those seen in females). The genes activated at this early 3 h time point suggested gene activation predominantly involved in transcription, BBB and endothelial function, and in inflammation [14]. Our study was exploratory and designed to examine if there are sex similarities and differences in gene expression. The study on females provides a better knowledge base for further research as well as increasing the chance of novel findings. Examining early gene expression is just a minor step in understanding the complex cascade of events that are initiated by ischemia-reperfusion [3], but we believe that it can provide a base for further investigation of differential gene and protein expression at later time points.

## Methods

We aimed at identifying early cerebral vascular responses to ischemic stroke in female rats and identifying potential new therapeutic targets. We also aimed at comparing the differential gene expression patterns observed in MCAs from female rats with those seen in males.

### Overview of study design and inclusion and exclusion criteria

The study was designed as a case-control study with the occluded and non-occluded MCAs (after tMCAO) as case groups and the MCAs from healthy rats (non-operated) as control group, and the primary study outcome was differential gene expression (gene expression in cases relative to controls). The sample size was set based on experience from earlier studies conducted in the lab. The experimental unit was individual animal, only animals surviving the operation until the 3 h’ time point were included and the confounding factors age, sex, estrous cycle and environmental factors were controlled for. The confounding factor sex was investigated as an explanatory variable. To reduce pain, suffering and distress, animals were operated under full anesthesia and sacrificed under sedation. The following exclusion criteria were applied during animal experiments: (i) Not satisfactory occlusion or reperfusion, (ii) no reduction in neuroscore, or (iii) death during or after the procedure; and during microarray or qPCR: (i) Not satisfactory purity or concentration of RNA. All inclusion and exclusion criteria were specified prior to the experiments, except for the satisfactory occlusion threshold which was specified during the experiments. Ten female rats and 6 male rats were excluded due to death during the procedure or failing to meet the exclusion/inclusion criteria. For the microarray, three samples were excluded due to poor RNA integrity and 1 sample was excluded due to poor amplification. For the qPCR, seven samples were excluded at this stage due to low RNA-concentration and 3 samples due to failed amplification (Fig. S1).

### Animals

12-week old female (*n* = 42) and male (*n* = 18) Wistar rats were obtained from Charles River (Charles River laboratories, Sulzfeld, Germany). After applying the inclusion and exclusion criteria, 23 female and 12 male rats remained (Fig. S1). Animals were housed in environmental enriched cages with a 12 h light/dark cycle (lights on 7 am-7 pm), climate- and humidity- controlled environment and with free access to food and water in the University of Lunds animal facility. Prior to initiating the experimental procedures, animals were subjected to an acclimatization period. All experimental procedures were performed in accordance with the ARRIVE guidelines, the ethical guidelines of the International Association for the Study of Pain regulations on animal welfare and the National Institutes of Health guidelines for the care and use of laboratory animals. The experimental procedures have been previously approved by the Institutional Animal Care and Use Committee of the University of Lund. The rats were randomly divided into control and tMCAO experiments. The female rats were monitored daily with vaginal smears for a minimum of two consecutive cycles prior to sacrifice to determine the estrous phase. Samples were collected with a saline soaked cotton swab, transferred onto microscope slides, air-dried and stained with hematoxylin-eosin. The samples were collected at the same time-point each day. Female rats that were in the high estrogen phase, proestrus, were not included to reduce variation due to hormone fluctuations. The methodology has been further described by Goldman et al. [37].

### Transient middle cerebral artery occlusion

MCA occlusion was performed by using the intraluminal filament technique [38]. Anesthesia was induced by 3.5% isoflurane in N_2_O:O_2_ (70:30) and maintained by continuous inhalation of 2.5% isoflurane in N_2_O:O_2_ (70:30). An arterial tail catheter was inserted to monitor blood gases and mean arterial blood pressure during the occlusion. A rectal thermometer connected to a homoeothermic blanket was used to maintain body temperature at 37 °C during the procedure. A laser Doppler probe (Perimed, Järfälla, Sweden) was fixed to the skull 6 mm laterally of the midline and 1 mm posterior to bregma. An incision was made in midline of the neck, exposing the carotid artery. The external carotid and common carotid were permanently ligated with sutures. A rubber-coated monofilament (Doccol Corporation, Redlands, CA, USA) was inserted through an incision in the common carotid artery through the internal carotid artery until a sudden drop in cerebral blood flow was observed in the area supplied by the MCA (as measured with Laser Doppler flowmetry). Animals with a minimum flow reduction of 50% were included in the study. The filament was secured by sutures and the surgical areas closed while the anesthesia was discontinued, and the animal allowed recovering. The method has been described in details before [21, 22].

Prior to reperfusion, the rats were evaluated with the 6-point neuroscore [39, 40]. Only animals showing a score of 3–4 in the test were re-anesthetized after 120 min and the filament removed, which resulted in an increase in cerebral blood flow (as measured with Laser Doppler flowmetry). Animals with a minimum flow increase of 30% were included in the study. The animals were then allowed to recover with free access to food and water for 3 h.

### Middle cerebral artery isolation

Three hours post reperfusion; the animals were euthanized with carbon dioxide and decapitation. The brain was immediately removed and both the right and left MCAs (distal part; length = 5 mm, diameter 0.2 mm) were carefully dissected out. The MCAs were carefully cleaned from surrounding connective tissue and blood, frozen on dry ice and stored in − 80 °C. Inclusion criteria were successful tMCAO operation as judged from the Laser Doppler analysis of regional cerebral blood flow (rCBF) and neuroscore evaluation. All animals fulfilling this survived the 3 h' until sacrifice. The subsequent handling and analysis of the removed MCA segments were semi-blinded meaning that the tissue and group were unknown to the analyst from this time point until the time where the data were analyzed.

### RNA-extraction

The extraction of RNA was performed with the same method for both the microarray and PCR. The RNA was isolated with the Nucleospin miRNA isolation kit (Machery-Nagel, Düren, Germany), following the manufactures instructions for extraction of total RNA. The artery-samples were first homogenized in in Lysing matrix D tubes containing 1.4 mm ceramic spheres (MP Biomedicals, CA, USA) and lysis buffer (ML buffer) from the NucleoSpin kit on dry ice in a FastPrep-24™ 5G instrument (MP Biomedicals, USA) with 3x20sec cycles.

After RNA extraction, the amount of RNA was quantified using a NanoDrop 2000 UV-Vis spectrophotometer (ThermoFisher Scientific, MA, USA). A ratio of sample absorbance at 260 nm and 280 nm in the range of 1.7 to 2.1 was accepted. The RNA which was to be used for microarray analysis was concentrated with a Scan Speed 32 speed vacuum concentrator (Labogene, Denmark). The concentration and quality of the concentrated RNA was determined with a NanoDrop ND1000 spectrophotometer (ThermoFisher Scientific, MA, USA).

### Whole genome microarray

Affymetrix whole-transcriptome expression profiling was processed by Swegene centre for integrative biology (SCIBLU) genomics, Affymetrix unit at Lund University, Sweden.

MCAs from 6 stroke females and 6 female controls were analyzed. The integrity of the RNA was measured with the Agilent Bioanalyzer (Agilent Technologies, CA, USA). From a total of 100 ng RNA, single stranded complimentary DNA (cDNA) was synthesized using primers containing a T7 promoter sequence. The single stranded cDNA was converted to double stranded DNA and used as a template for in vitro transcription, producing complimentary RNA (cRNA) [41]. After purification, sense-strand cDNA was synthesized and purified. The sense strand cDNA was fragmented and labeled and loaded onto Affymetrix GeneChip rat gene 2.0 ST arrays (ThermoFisher Scientific, MA, USA). This was followed by hybridization for 16 h at 45 °C in an Affymetrix Gene Chip Hybridization 645 oven. The array was scanned using the Affymetrix GeneChip scanner 3000 7G.

### Complimentary DNA synthesis and quantitative PCR

MCAs from 5 female stroke rats, 6 male stroke rats and 6 controls of both sexes were analyzed. After RNA extraction, the amount of RNA was quantified using a NanoDrop 2000 UV-Vis spectrophotometer (ThermoFisher Scientific, MA, USA). cDNA was synthesized using the RT2 First Strand Kit (Qiagen, Germany) according to the manufacturer’s protocol.

The QuantStudio 12 K Flex real-time PCR system (ThermoFisher Scientific, MA, USA) was used for the qPCR. Taqman gene expression assays for *Ccl2* (Rn00580555_m1), *Olr1* (Rn00591116_m1), *Adamts4* (Rn02103282_s1), *Serpine1* (Rn01481341_m1), *S1pr3* (Rn01757498_m1), *Socs3* (Rn01470502_g1), *JunB* (Rn00572994_s1) and *Fosl1* (Rn00564119_m1) were purchased from ThermoFisher Scientific, MA, USA.

The qPCR was performed in a 10-μl reaction volume containing TaqMan 2× universal PCR master mix (ThermoFisher Scientific, MA, USA), 20× TaqMan gene expression assay, RNase-free water and 2 μl cDNA using the QuantStudio 12 K Flex real-time PCR system (ThermoFisher Scientific, MA, USA) with ROX as a passive reference. A no-template control with RNase-free water instead of cDNA was used as negative control for all TaqMan gene expression assays. An inter-plate control for all TaqMan gene expression assays was used to control the thermal cycling between plates. *ActB* (Rn00667869_m1) and *Gapdh* (Rn01775763_g1) acted as housekeeping genes. All TaqMan gene expression assays were pipetted in triplicates for each sample.

### Analysis and statistics

#### Analysis of microarray data

Basic Affymetrix GeneChip analysis and experimental quality control were performed using the Expression Console Software (v1.1.2), and the Robust Multi-array analysis method was used for probe summarization and data normalization (quantile normalization and log transformation). Data filtration was done for probe sets having a value less than the median values of the negative control in 80% of total samples.

Significance analysis of microarray was performed using the TMEV software (v4.0). Differentially expressed genes with a false detection rate (q-value) of zero were selected and single probe sets with more than one annotation were excluded. For downstream analyses, we performed enrichment analyses using the PANTHER Classification System [42–44]. Enriched PANTHER pathways and protein classes (v15), Reactome pathways (v65) and GO biological process terms (released 2020-02-21) were identified using Fisher’s Exact Test and the *p*-values were adjusted for multiple testing using the conservative Bonferroni correction. *P* < 0.05 was considered statistically significant.

For the biological process GO terms, only the most specialized term within each hierarchical group was included and GO terms with less than 30 annotated genes from the list of differentially expressed genes were excluded. For the PANTHER protein classes and Reactome pathways, only the least specialized protein class/pathway within each hierarchical group was included (cut-off: at least 10 annotated genes). On the contrary, only the most specialized pathway within each hierarchical group was included for the PANTHER pathways (cut-off: at least 5 annotated genes). Subsequently, overlap between overrepresented GO terms for the differentially expressed genes in the occluded MCAs and non-occluded MCAs both compared with control MCAs were identified.

To explore interactions between selected gene products, we utilized the Search Tool for the Retrieval of Interacting Genes/Proteins (STRING) database v11.0 [45]. Only experimentally determined and database curated *Rattus norvegicus* protein-protein interactions were used to create the networks. We defined a cluster as a network formed by at least 5 interacting proteins.

#### Gene selection and GO term categorization

Based on the results from the microarray, eight genes were selected for validation with qPCR. *Ccl2*, *S1pr3*, *Socs3*, *Serpine1*, *JunB* and *Fosl1* were selected due to occurrence in biological processes that were significantly enriched in the GO analysis in combination with analysis of fold change and review of literature. *Adamts4* was selected due to a very high relative expression, and *Olr1* was selected due to the availability of previous data in the same model in hypertensive and normotensive males. In addition to qPCR, we categorized the selected genes into preselected GO biological process terms using the PANTHER Classification System [42–44] and the statistical software R (v4.0.2) to examine their involvement in specific biological processes.

#### qPCR calculations

In the PCR-validation, the mean computed cycle threshold (CT) for the triplicates of each sample was calculated. The results were normalized against the inter-plate control. The mean CT-value from the housekeeping genes ActB and Gapdh were subtracted from the mean CT value of the target gene to be able to compare samples that contained a differing amount of total-RNA. This relative value is hereafter referred to as delta-CT (dCT). For each gene, dCT values more than 3 standard deviations away from the mean were considered outliers and excluded (only one observation was considered an outlier across the datasets). Subsequently, the effects of sex (males and females) and experimental group (control MCAs, non-occluded MCAs and occluded MCAs) on dCT were investigated by means of linear mixed-effect models with restricted maximum likelihood using the statistical software R (v4.0.2) and the nlme R package (v3.1.148) [46]. Sex and experimental group were fitted as fixed effects (fixed effects were omitted from the model if not reaching significance) and animal ID as a random effect to consider individual animal differences. To assess normality and equality of variance, we created and examined Q-Q plots and residuals versus fitted values plots, respectively. These data are presented using boxplots and statistical significance was set at *p* < 0.05. Since a higher dCT value corresponds with a lower expression, the axes in the figures were reversed to offer a more intuitive reading of the figures.

#### Cross-analysis with findings from Grell et al. [20]

To reveal sex similarities between males and females at a larger scale, we compared the findings from the current study with findings obtained by Grell et al. [20]. Here, they investigated differential expression of genes in the occluded MCAs compared with the non-occluded MCAs in WKY male rats. They used the same stroke model (tMCAO), the rats were the same age (12-weeks) and the samples were processed by the same center using the same method (SCIBLU). Even though the groups are similar in many ways, some of the variations observed might be due to strain differences rather than sex differences. Therefore, we decided to focus solely on the overlapping differentially expressed genes.

## Supplementary Information

**Additional file 1.**

**Additional file 2.**

## Data Availability

The datasets generated or analysed during the current study are available in the Gene Expression Omnibus (GEO) repository, GSE162072, and in the published article [and its supplementary information file ‘Rehnstrom_et_al_supplementary_files.xlsx’].

## Abbreviations

*ActB*: Actin B
*Adam*: ADAM metallopeptidase domains
*Adam8*: ADAM metallopeptidase domain 8
*Adamts*: A disintegrin and metalloproteinase with thrombospondin motifs
*Adamts4*: A disintegrin and metalloproteinase with thrombospondin type 1 motif, 4
AP-1: Activator protein 1
ATF-2: Activating transcription factor–2
BBB: Blood-brain barrier
*Ccl*: Chemokine (C-C motif) ligand
*Ccl2*: Chemokine (C-C motif) ligand 2
*Chi3l1*: Chitinase-3-like protein 1
CT: Cycle threshold
*Cxcl*: Chemokine (C-X-C motif) ligand
dCT: Delta cycle threshold
ECM: Extracellular matrix
ELK-1: ETS transcription factor
*Fkbp3*: FK506 binding protein 3
*Fosl1*: Fos-like antigen 1
*Gapdh*: Glyceraldehyde 3-phosphate dehydrogenase
GO: Gene ontology
*Icam1*: Intercellular adhesion molecule 1
*Il*: Interleukin
*Il-6*: Interleukin-6
JNK: Jun amino-terminal kinases
*JunB*: Jun B proto-oncogene
*Nfkb1*: Nuclear factor of kappa light polypeptide gene enhancer in B-cells 1
*Rela*: Nuclear factor kappaB subunit p65
MCA: Middle cerebral artery
*Mmp*: Matrix metallopeptidase
*Mmp19*: Matrix metallopeptidase 19
*Olr1*: Oxidized low-density lipoprotein receptor 1
PANTHER: Protein ANalysis THrough Evolutionary Relationships
pERK1/2: Phosphorylated extracellular signal-regulated kinase 1 and 2
pJNK: Phosphorylated c-jun N-terminal kinase
pPKC: Phosphorylated protein kinase C
pPKCδ: Phosphorylated protein kinase C-δ
*Prdm4*: PR domain zinc finger protein 4
qPCR: Quantitative real-time polymerase chain reaction
rCBF: Regional cerebral blood flow
ROS: Reactive oxygen species
*Runx1*: Runt-related transcription factor 1
S1P: Sphingosine 1-phosphate
*S1pr3*: Sphingosine 1 phosphate receptor 3
SAPK: Stress-activated protein kinases
*Serpine1*: Serine protease inhibitor, clade E, member 1
*Socs3*: Suppressor of cytokine signaling 3
*Stat3*: Signal transducer and activator of transcription 3
STRING: Search Tool for the Retrieval of Interacting Genes/Proteins
*Ttc39b*: Tetratricopeptide repeat domain 39B
tMCAO: Transient middle cerebral artery occlusion
*TNFα*: Tumor necrosis factor α
VSMC: Vascular smooth muscle cell
WKY: Wistar Kyoto
*Zc3h12a*: Zinc finger CCCH type containing 12A
