# Supplementary material for: Transcriptome profiling revealed early vascular smooth muscle cell gene activation following focal ischemic stroke in female rats – comparisons with males
Source: BMC Genomics. 2020 Dec 9;21:883. doi: 10.1186/s12864-020-07295-2 (PMC7726885; doi:10.1186/s12864-020-07295-2)
Supplement: Supplementary file 1 — Additional file 1. [file 12864_2020_7295_MOESM1_ESM.pdf]

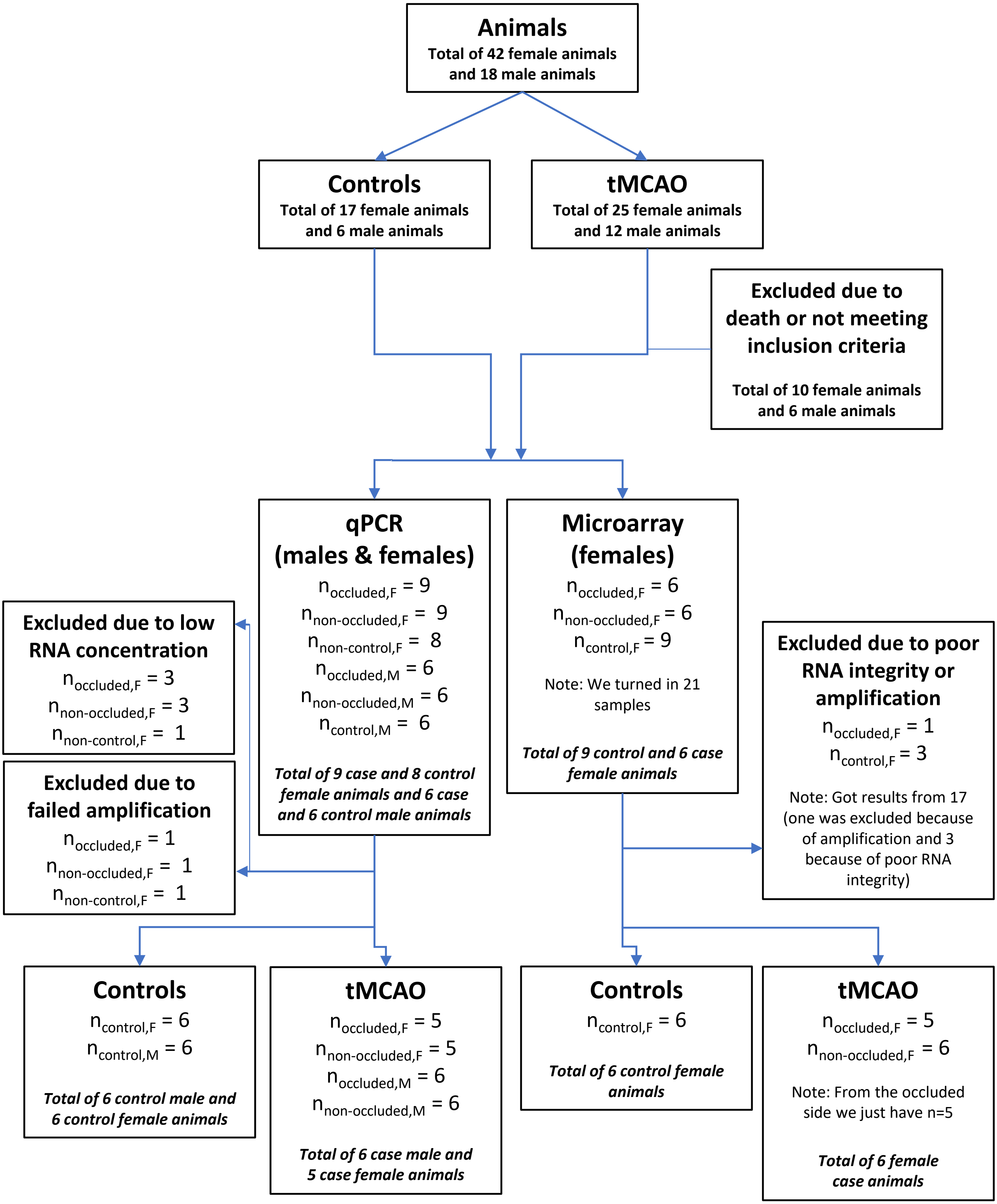

A total of 60 animals were initially included in the study

After applying the inclusion and exclusion criteria, a total of animals (23 females and 12 males) remained to be analyzed in the study.
12 animals by microarray and 23 by qPCR.
